# La mirada del alumnado de medicina hacia la tutorización longitudinal en atención primaria

**DOI:** 10.1016/j.aprim.2025.103394

**Published:** 2026-01-16

**Authors:** Yoseba Cánovas Zaldúa, Francesc Alòs, Silvia Güell Parnau, Raquel Gayarre Aguado

**Affiliations:** a Equipo de Atención Primaria Passeig de Sant Joan, Gerencia de Atención Primaria y a la Comunidad Barcelona Muntanya-Dreta, Institut Català de la Salut (ICS), Barcelona, España; b Departamento de Medicina, Universidad Autónoma de Barcelona (UAB), Barcelona, España; c Fundació Institut Universitari per a la recerca a l’Atenció Primària de Salut Jordi Gol i Gurina (IDIAPJGol), Barcelona, España; d Equipo de Atención Primaria Montcada i Reixach, Gerencia de Atención Primaria y a la Comunidad Barcelona Muntanya-Dreta, Institut Català de la Salut (ICS), Montcada i Reixach, Barcelona, España; e Equipo de Atención Primaria Encants, Gerencia de Atención Primaria y a la Comunidad Barcelona Muntanya-Dreta, Institut Català de la Salut (ICS), Barcelona, España

La Medicina Familiar y Comunitaria (MFyC) constituye una disciplina central en los sistemas sanitarios, al integrar una atención centrada en la persona, la longitudinalidad asistencial y una visión comunitaria. Sin embargo, su peso en la formación de grado en España continúa siendo limitado, lo que puede influir en la percepción de los estudiantes sobre el rol de la atención primaria (AP) en el sistema^1^, ^2^. En este contexto, los modelos de tutorización longitudinal, ampliamente desarrollados en otros países, ofrecen una oportunidad para acercar de manera temprana y continuada al estudiantado de medicina la práctica clínica en AP, favoreciendo la adquisición de competencias y el desarrollo de una identidad profesional^3^, ^4^.

Con el objetivo de explorar la percepción de los futuros médicos sobre la implantación de un modelo de tutorización longitudinal en AP, se llevó a cabo un estudio exploratorio secuencial durante el curso académico 2023-2024 en la Universidad Autónoma de Barcelona. Participaron 365 estudiantes de primer curso del grado de medicina matriculados en la asignatura «Práctica Clínica Asistencial I», quienes debían cumplimentar un portafolio reflexivo acerca de las prácticas en el centro de salud que incluía cuatro preguntas abiertas sobre la propuesta de modelo. El análisis del contenido permitió agrupar las respuestas en categorías temáticas y cuantificar su frecuencia.

De los 354 portafolios recogidos (96% de la muestra), el 98% consideró adecuada la propuesta de tutorización longitudinal. Entre los aspectos más valorados destacaron la posibilidad de establecer un vínculo continuado con el tutor (44%), la motivación por aprender en un entorno de AP (18%) y la personalización del aprendizaje (6%). En relación con los contenidos deseados, el alumnado expresó interés en desarrollar actividades asistenciales básicas (19%), adquirir habilidades de comunicación (15%) y explorar la relación médico-paciente (13%).

Respecto a la organización de la docencia práctica, el 92% manifestó el deseo de aumentar los días de estancia en el centro de salud, siendo la preferencia mayoritaria una jornada al mes durante todo el grado. Finalmente, los criterios de evaluación más mencionados fueron la predisposición del alumno a aprender (23%), su actitud proactiva (21%) y aspectos de profesionalismo médico (17%).

Estos resultados, reflejan una elevada aceptación y un interés explícito en intensificar la experiencia práctica en AP desde los primeros cursos del grado. La percepción favorable coincide con la literatura internacional, que ha señalado el impacto positivo de la tutorización longitudinal en el aprendizaje de los estudiantes, aumentando el compromiso y el conocimiento hacia la especialidad y mejorando su nivel de elección al terminar el grado^5^, ^6^.

Este trabajo constituye la primera aproximación en nuestro entorno sobre la visión del alumnado respecto a una propuesta de modelo de tutorización longitudinal en AP.

En conclusión, los estudiantes valoran de manera muy favorable la posibilidad de contar con médicos de familia que realizaran una tutorización longitudinal en sus prácticas en AP. Identifican la relación continuada entre el tutor y el estudiante, la motivación por aprender en un entorno de AP y el desarrollo de competencias profesionales como aspectos clave, al tiempo que demandan una mayor frecuencia de días de prácticas en el centro de salud. La implementación de este modelo podría suponer una innovación docente con impacto positivo en la formación de los futuros estudiantes de medicina y en la atracción hacia la MFyC.

## Financiación

El presente estudio ha contado con una Beca XB otorgada por la Gerencia de Atención Primaria a la Comunidad Barcelona Muntanya-Dreta del Institut Català de la Salut (ICS).

## Conflicto de intereses

Los autores declaran no tener conflicto de intereses.
